# Moisture buffering and mould growth characteristics of naturally ventilated lime plastered houses

**DOI:** 10.14324/111.444/ucloe.1988

**Published:** 2024-09-10

**Authors:** Vismaya Paralkar, Rashmin Damle

**Affiliations:** 1 CEPT University, Ahmedabad, India

**Keywords:** lime plaster, hygrothermal simulations, mould growth, surface relative humidity conditions

## Abstract

Lime plaster is a sustainable building material that can be an effective passive cooling strategy. The moisture buffering quality of lime causes adsorption and desorption of moisture which moderates the indoor relative humidity. Its vapour permeability is also influential in moisture transfer across the building envelope. Lime plaster also has a self-healing quality which prevents the formation of inner cracks. Moreover, its strength increases with time leading to a longer life span. In old structures, an important function is the breathability of ceilings and walls. Hence, it is often used in conservation projects where it improves the appearance and durability of old buildings. Often organic additives employed to impart certain qualities to the lime mortar/plaster led to mould growth. Mould growth degrades indoor air quality, and the occupant health is compromised. To avoid mould-related problems, it is necessary to understand the behaviour of lime plaster with respect to the indoor relative humidity and surface moisture content. This paper focuses on the hygrothermal performance of lime plaster in naturally ventilated residential spaces. Surveys were carried out in 45 traditional buildings in Ahmedabad in India with measurements of ambient variables, such as temperature, relative humidity, wall moisture content, etc. The mould growth patterns of these spaces are related to the measured variables and wall characteristics. Hygrothermal simulations of some spaces were also carried out to observe the moisture buffering of lime plaster. Experimental observations were then compared to simulation results to see if the predictions of the hygrothermal models were realistic.

## Introduction

The relative humidity (RH) of air in a building determines its energy performance, comfort and indoor air quality [1]. Moisture buffering characteristics of wall materials can modulate indoor humidity by absorbing/desorbing the moisture present in the indoor air [2]. Woloszyn and Rode demonstrated that the use of hygroscopic materials in the envelope and a well-controlled heating, ventilation and air conditioning (HVAC) system could reduce the cooling and heating energy consumption by 5% and 30%, respectively [2]. They achieved energy efficiency in buildings by combining moisture buffering with RH-sensitive ventilation. By employing hygroscopic gypsum and wood fibre materials, an energy saving potential of 25–30% for temperate and semi-arid climates was observed [3]. The hygroscopic interaction between the wall surface and room air affected the air temperature by 2–3 °C [4]. Due to moisture buffering, up to 20% reduction in heating energy was observed in different climate zones of India [5].

Apart from the energy aspect, RH also affects the concentration of noxious gases in the air as it alters the rate of off-gassing in the building materials. Due to microbial interactions, odorous and irritant substances or allergens are emitted from damp materials. The presence of moisture is a cause of deterioration inside buildings [6] while affecting both sensible and latent heat loads [7]. In addition to thermal discomfort, RH coupled with high temperature may also lead to adverse health effects such as exhaustion and heat stroke, and can possibly prove to be fatal. Its indirect effect on human health can lead to allergic incidences and respiratory diseases [8]. Several health issues such as asthma and respiratory disorders are associated with dampness in buildings.

The fungal growth on building surfaces is highly influenced by the RH of indoor air. Twenty-one different types of building materials were studied [9] to understand the influence of temperature and RH on the metabolism and growth of eight different micro-fungi. Under constant temperature conditions, with RH up to 95% and water activity at 0.95, *Penicillium*, *Aspergillus* and *Eurotium* overgrew other indoor fungi. The minimum water activity for fungal growth in materials susceptible to mould growth is 0.78–0.8 [9]. For the same water activity, favourable conditions for mould occur at 10 °C under 80–90% RH and at 5 °C for RH >90%. The four sources of dampness and moisture in the building are leakage of rain and snow into the building construction or moisture from the ground, moisture from occupants and their indoor activities, and water leakage [10]. Moreover, the problem of even small water leaks has a significant impact if the moisture buffering capacity of the material is low. Therefore, the moisture transfer/buffering capacity of building materials plays a vital role in regulating the indoor RH by moisture transfer through the building envelope.

Lime is an environmentally friendly material that has been largely forgotten in contemporary construction practices. Before the advent of Portland cement, lime mortar and lime plaster were widely used as binding and finishing materials. The use of lime plaster in the Ellora caves [11] and the baked brick walls at Karvan [12] in India dates back to the 6th century AD. Lime plaster mixed with hemp, dolomite or cannabis was applied in different layers for longevity [13]. Lime has lower embodied energy [14] and excellent moisture buffering capacity [15]. Liuzzi and Stefanizzi reported a reduction in humidification and dehumidification energy requirements with lime-stabilised bentonite clay [16]. In comparison to cement mortars, lime-based mortars had three times more moisture buffering capacity [17]. Post-occupancy analysis of lime-plastered spaces in a warm and humid climate zone showed 5–15% variation in indoor humidity levels than the outdoor humidity levels for studied spaces under a lime plastered dome and a vault [1]. Lime plaster is also vapour-permeable, that is, useful in ‘breathing wall’ construction [18]. It is recyclable, involves nontoxic chemicals for manufacturing, and its production can be downscaled as needed. When used as an internal render, it improves indoor air quality by absorbing low amounts of carbon dioxide [19] and regulating indoor RH for a prolonged period [20]. Lime avoids the problems of decay and dampness by allowing the building envelope to breathe unlike several modern nonporous materials [21]. The rain exposure impact is low on hygrothermal performance of a capillary closed material such as mineral plaster. But the impact is significant on the mould index for a lime plaster assembly [22]. Lime plaster has higher capillary action and its moisture content is vastly dependent on its exposure to driving rain [23].

Variations in the location of humidity sources and room ventilation rates give rise to pockets of high RH. The average RH throughout the building should be maintained between 40% and 60%.

The main objective of this work was to study the mould growth propensity of naturally ventilated lime-plastered spaces. To this end a three-tier methodology has been employed:

1. Survey of naturally ventilated residential spaces in order to correlate the observed mould growth in terms of the building characteristics such as coatings on the wall, ventilation, the level of water activity inside the space and the function of the space, sunlight and clutter near the walls.
2. Hygrothermal simulations to assess the capability of the effective moisture penetration depth (EMPD) model [24] using EnergyPlus [25].
3. Experiments to study the onset of mould growth in lime plaster samples kept at different RH levels.

### Survey of naturally ventilated residential spaces

Two different types of naturally ventilated lime-plastered houses were surveyed in the city of Ahmedabad. Ahmedabad falls in the hot–dry climate zone of India. The heritage city of Ahmedabad includes thousands of Pols, a dense cluster of residences belonging to people of the same caste, religion and occupation. For more than 300 years, these neighbourhoods have been popularly known as Pol houses [26].

**Figure 1:**
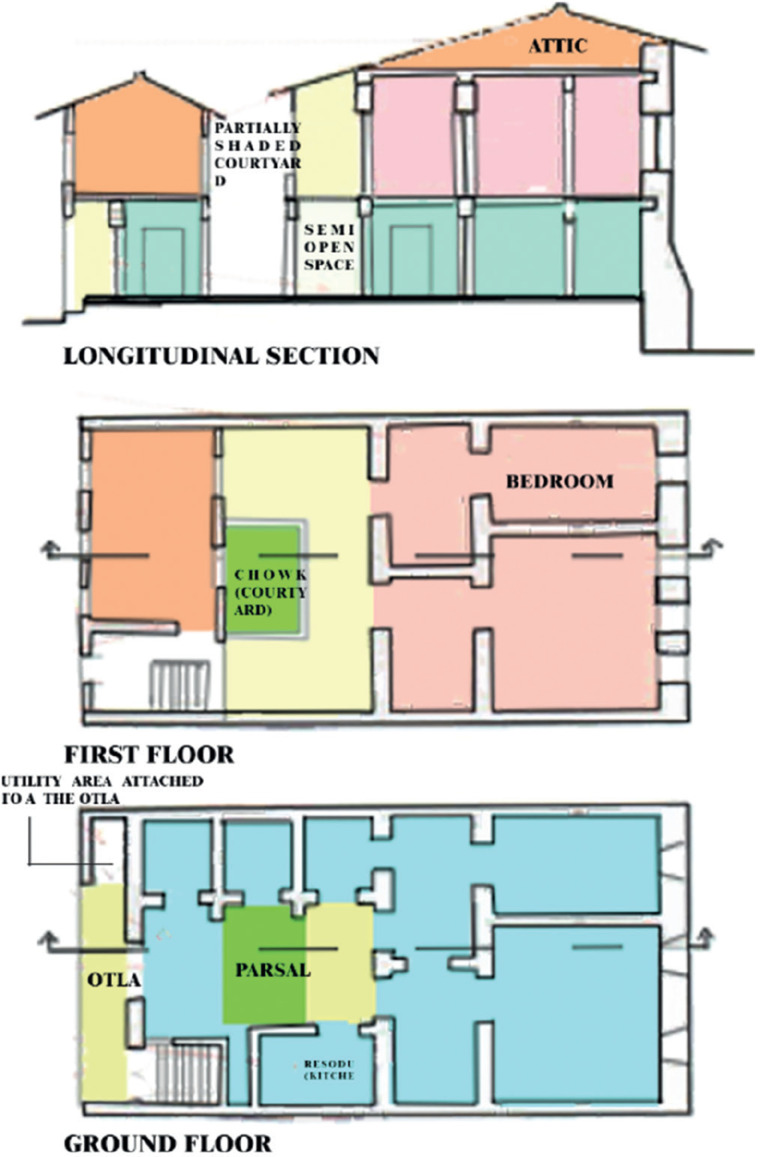
Details of a Pol house, Ahmedabad (Source: [27]).

Figure 1 shows the passive strategies in the design of Pols are prominent: protecting the inside spaces from direct heat gains include mutual shading, thick walls, long shared walls, central courtyards, multi-storey structures, narrow lanes, dense clusters, etc. The otla outside the house is the open space that is usually used to dry clothes in some houses. Attached to the otla is a small, dedicated space for washing utensils and toileting. The construction of the house is a timber framework with brick masonry and wall surface finish of lime plaster. Forty-five spaces were surveyed in Pols which had lime plaster as the rendering material with characteristics mentioned in the Appendix. The selection of these spaces was based on the type of wall finishes used [either lime plaster or plastic paint (waterproof paint)], the typology of the house and accessibility.

**Figure 2:**
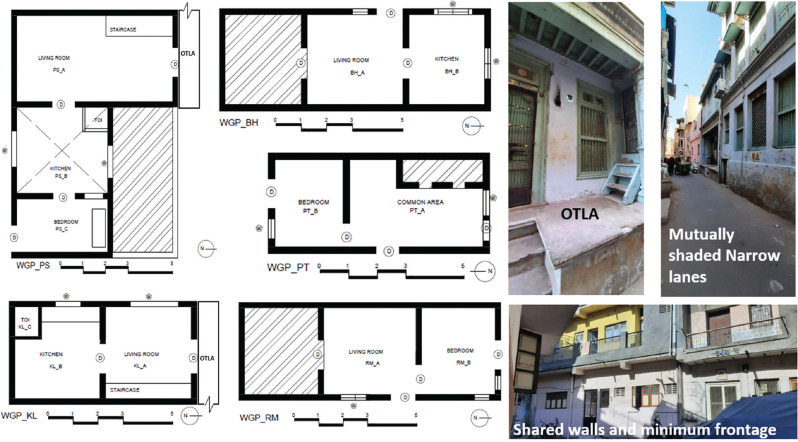
Pol houses.

**Figure 3:**
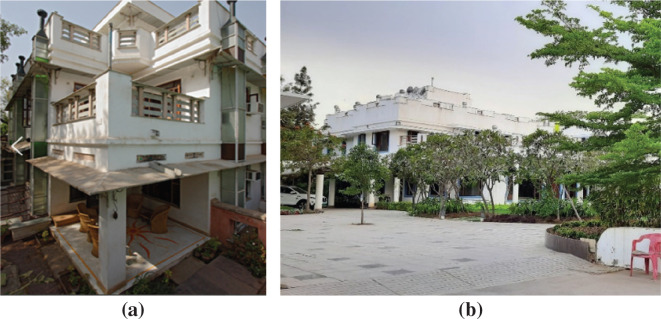
(a) Girikunj residence (Source: Abhikram Architects); (b) RSR residence.

Detached lime-plastered residences were also studied (Fig. 2). The Girikunj house (Fig. 3a) is a residential bungalow [28], designed using passive design strategies and traditional materials and methods. The ventilation system is well designed using glass exhaust shafts on the periphery. These shafts collect hot air from the small grill outlets in the spaces when the windows are closed. Air vents attached to it exhaust this air to the outside. Lime is employed for plastering, wall wash and mortar. It consists of additives such as Gur (jaggery), Gugal (*Commiphora wightii*) (Gugal water is used in lime plaster as an additive to enhance the plaster’s water proofing qualities) [29] and Methi (fenugreek) for improving binding and waterproofing.

The RSR house (Fig. 3b), a bungalow designed by Abhikram Architects [28], was constructed using lime mortar and lime plaster. The type of lime plaster used is Marmarino lime plaster, which gives a marble-like finish. The walls were not coated with paint. Mechanical ventilation is provided with the help of exhaust fans and air vents throughout the space.

**Figure 4:**
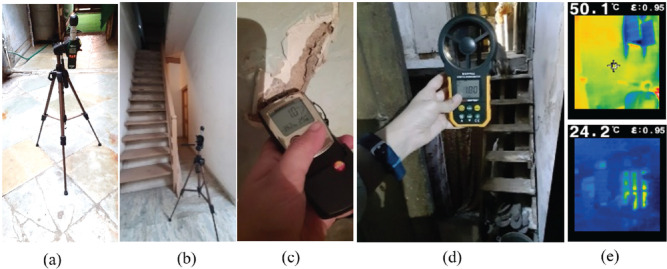
Site measurements using several instruments. See Table 1 for instrument details.

For the selected spaces, the function of and typical activities within each space were also noted. A layout of the space was created considering the wall thickness, opening area and adjacent spaces. Detailed architectural plans were drafted which showed the wall thickness, openings and adjacent spaces for each house. The volume is calculated from the measurements taken for the floor plans. Surveys were carried out in the afternoon from 2 pm to 4 pm when the outdoor temperatures were relatively high. Measurements were taken on every other day from 25 December 2019 to 16 March 2020. This resulted in around 85–90 readings for each space over the measurement period with a total of 3800 data points. Point in time measurements (Fig. 4), including outdoor air temperature, RH, globe temperature for calculating the mean radiant temperature (MRT), and air velocity, were noted for each space. Similarly, the inside air temperature, RH, globe temperature and air velocity at the centre of the space were measured. For measuring the inside surface temperatures of all the walls, the emissivity of the thermal gun was set to 0.95, which is the typical value of the emissivity of lime plaster. The surface temperature of the ceiling and floor was also noted.

Table 1 gives details of the measured parameters, their location and the respective instruments used in Fig. 4.

**Table 1.** List of instruments and their measured parameters

Instrument used	Parameter measured	Measurement location	Specifications
Heat Stress WBGT Meter (Extech HT30, Extech Instruments, USA) Fig. 4a	Outside air temperature, relative humidity, black globe temperature	Outside the survey space at a height of 1100 mm from the ground	Measuring range: Wet bulb globe (WBG) temperature – 32 °F to 122 °F (0–50 °C) Humidity: 0 to 100% RH Accuracy: WBG temperature – ±4 °F/2 °C Humidity – ±3% RH
Heat Stress WBGT Meter Fig. 4b	Inside air temperature, relative humidity, black globe temperature	At the centre of the survey space at a height of 1100 mm from the floor finish level	
Vane Anemometer (PEAKMETER MS6252A0, China) Fig. 4d	Air velocity	1. Near the globe thermometer, perpendicular to three planer axes 2. Perpendicular to the vertical plane of the openings inside a space	Measuring range: 0.40∼30.0 m/s Accuracy: ±(2.0% reading + 50)
Thermal Gun (FLIR TG165, USA) Fig. 4e	Surface temperature	Inside and outside exposed surfaces of all the walls surrounding the space (including the ceiling and floor)	Measuring range: 0.40∼30.0 m/s Accuracy: ±(2.0% reading + 50)
Testo 606-2 Moisture Meter (38767439/711, Testo, UK) Fig. 4c	Moisture content and surface relative humidity	Moisture content inside lime plaster and surface relative humidity near it	Measuring range: −10 to +50 °C 0 to 100% RH Accuracy: ±0.5 °C/0.1 °C ±2.5% RH (5 to 95% RH)
HOBO U10 Temperature and Humidity Logger (Onset, USA) Fig. 7 (Inset)	Temperature and RH logger	Inside the experiment jars to log whether the desired RH levels are kept constant	Measuring range: Temperature: 20 °C to 70 °C (−4 °F to 158 °F) Humidity: 25 to 95% RH Accuracy: Temperature: ±0.53 °C from 0° C to 50 °C (±0.95 °F from 32 °F to 122 °F) Humidity: ±3.5% from 25% to 85% over the range of 15 °C to 45 °C (59 °F to 113 °F) ± 5% from 25% to 95% over the range of 5 °C to 55 °C (41 °F to 131 °F)

### Simulating the studied spaces with the EMPD model

**Figure 5:**
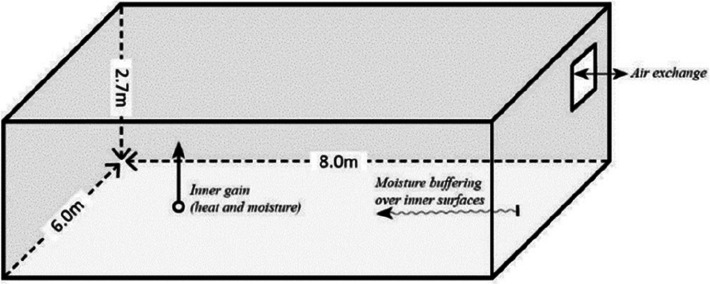
BESTEST geometry (Source: [30]).

Before simulating the surveyed spaces, preliminary simulations are worked out with the Building Energy Simulation Test (BESTEST) geometry [30]. These simulations are carried out with both the EMPD [31] and thermal-only, that is, the conduction transfer function (CTF) models of EnergyPlus [24]. The EMPD model considers the moisture absorption and desorption at wall surfaces, while the thermal-only model ignores it completely. The purpose of employing these two models is to check whether moisture absorption and desorption at the wall surfaces are captured by the EMPD model. Several authors [3,25,31,32] have employed the BESTEST geometry in their hygrothermal studies. Figure 5 shows the BESTEST geometry, which is a single-zone space of 8 m × 6 m × 2.7 m. The space has a moisture source of 500 g/h from 9 am to 5 pm and a constant air change rate of 0.5 air changes per hour for Ahmedabad weather [30]. The walls are assumed to be made of 230 mm thick brick masonry. For the inner wall surfaces finishing materials such as gypsum plasterboard, plywood and lime plaster are considered. The properties of these materials were considered from the EnergyPlus database [33].

EMPD is a simplified model which considers the moisture absorption and desorption at wall surfaces. It assumes a thin layer of air near the wall surface which is dynamic and exchanges moisture in the air in cyclic pulses of air moisture. EMPD gives a reasonable approximation of reality for short periods when there is no net moisture storage [34]. The EMPD model is employed in this work because it needs lesser information on hygric properties of materials in comparison to the detailed the Combined Heat and Moisture Transfer (HAMT) model of EnergyPlus [24].

**Figure 6:**
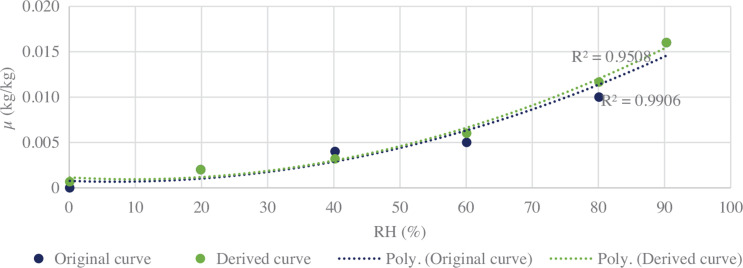
Best-fit sorption curve for lime plaster.

It is a challenge to derive a universal sorption curve for lime plaster as its composition is site-specific and varies from region to region. The sorption curve was derived from the moisture content values of building materials available in the literature [35]. The sorption coefficients were then derived through these moisture content values by a close-fitting curve as shown in Fig. 6. The difference observed between the curves is very minuscule with a maximum difference of 0.002 μ (kg/kg). These values were then fed into the EMPD model to carry out simulations. After carrying out hygrothermal simulations for BESTEST geometry, a sample surveyed space is simulated with the EMPD model. The change in MRT, RH and surface temperatures were observed through simulations. This further helped to predict the trend of the hygrothermal behaviour of lime plaster in that space throughout the year. The weather files for simulation are taken from EnergyPlus weather data^1^.

#### Studying the onset of mould growth in lime plaster samples

**Figure 7:**
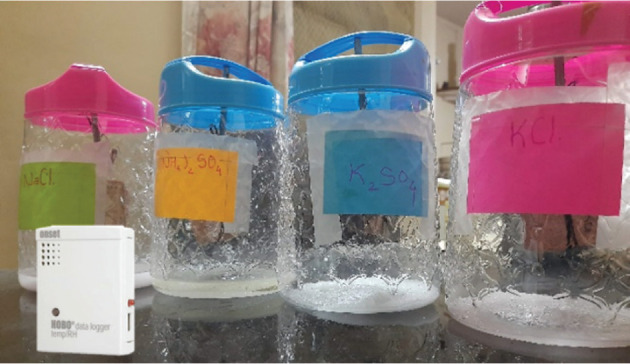
Jars containing different salt solutions and (inset) the HOBO U10 Temperature and Humidity Logger.

To understand the onset of mould growth on lime plaster, an experiment similar to [36] was carried out. Different RH levels were maintained in containers by different salt solutions. Table 2 shows the different salts and the quantity of salt and water to maintain the RH level inside the container. For this experiment, four plastic containers were filled with different salt solutions as shown in Fig. 7. Preliminary trials were carried out by trial and error to fix the amount of salt and water to obtain the specific RH value. The temperature and RH loggers (Hobo U10, Onset, USA) were attached on the inner side of the lid of the jars to ensure constant RH inside the containers.

**Table 2.** RH achieved for different salt compositions

Name of salt	Composition of salts	RH gained	Quantity (g)	Water quantity (g)
Sodium chloride	NaCl	75%	53.33	27.28
Ammonium sulphate	(NH_4_)_2_SO_4_	80%	53.33	30.00
Potassium chloride	KCl	86%	30.00	10.00
Potassium sulphate	K_2_SO_4_	99%	53.33	18.19

**Figure 8:**
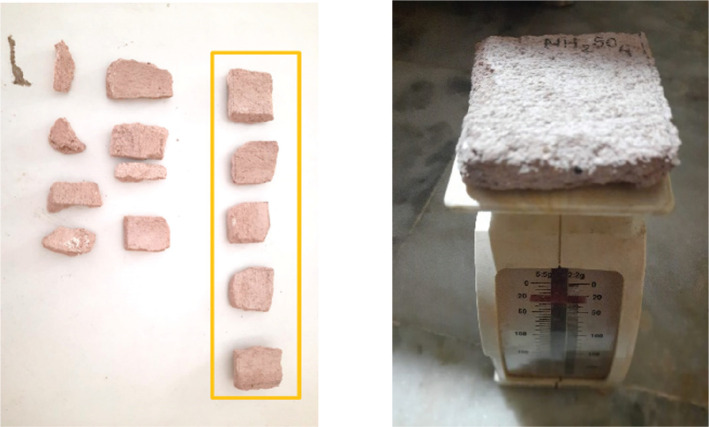
Lime plaster samples used for experiment.

The samples of lime plaster (see Fig. 8) contained one part of lime, one and a half parts Surkhi (burnt red-clay brick powder) and two parts of fine aggregate sand in the mixture. Additives such as jaggery water and Gugal water were also mixed to improve the waterproofing qualities of the mixture. The jars were kept in a space where the temperature was between 25 °C and 30 °C and the maintained RH levels were logged for 4 weeks at an interval of 5 min. Once the desired RH levels were achieved, lime plaster samples of equal size (40 mm × 40 mm × 10 mm), weight and composition were introduced into the containers. The samples remained in closed containers at the respective RH value maintained by the specific salt solution. This experiment was carried out to identify the onset of mould growth.

## Results and discussion

### Simulation results with the BESTEST geometry

**Figure 9:**
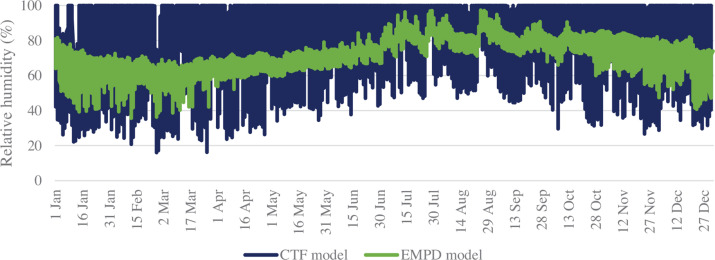
RH of space with CTF and EMPD models.

Figure 9 shows the RH in the space with lime plaster as the finish material for inside surfaces. The contrast in the RH levels of thermal-only (CTF) and EMPD models is visible. In the case of the CTF model, the RH levels go up to 100%. While with the EMPD model, the RH levels are maintained between 39% and 95%. Thus, the EMPD model [24] captures the moisture buffering effect of lime-plastered wall surfaces.

**Figure 10:**
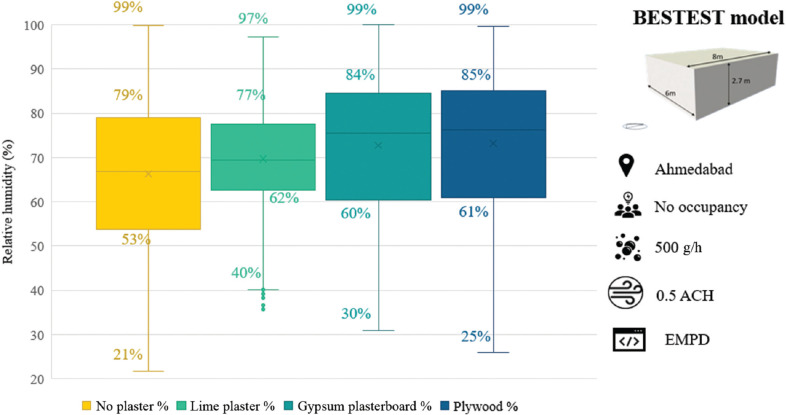
Indoor RH in different materials.

Simulations with the EMPD were also carried out with gypsum plasterboard and plywood as inner surface finishes. Figure 10 shows the box plot of RH values for the different cases. Amongst the different surface finish materials, lime plaster showed the least variation in RH levels and the least amplitude. In the cases of gypsum plasterboard, plywood and no plaster material the maximum RH is 99%. While the maximum RH with lime plaster was about 97%, RH values with lime plaster and plasterboard do not drop below 30% for the given conditions. Also, 50% of the time, RH with lime plaster was in the range of 62–72%. The RH values were lower during moisture increase and higher during moisture reduction with lime plaster. The above confirms the better performance of lime plaster over the rest of the finish materials.

### Simulation results of surveyed sample spaces

Annual hygrothermal simulations with the EPMD model were carried out for two spaces – PT_B and PS_A. The geometry of these spaces was recreated for the simulations. Physical parameters similar to the actual space were created except for the lime composition which was unknown. Spaces PT_B and PS_A had an occupancy of 1 and 3, respectively, and there was no external source of the moisture. These spaces were selected because they showed substantial mould growth to check simulation predictions. The performance in terms of MRT and indoor RH were observed throughout the year.

**Figure 11:**
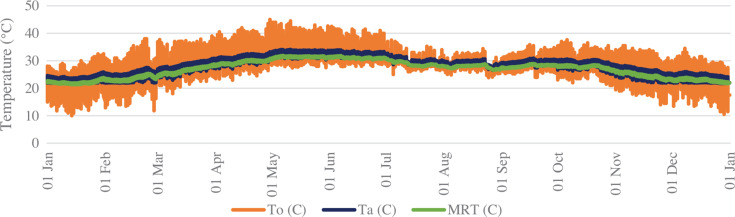
Outdoor, indoor and mean radiant temperature in space PT_B.

Figure 11 shows the temperature variations observed from outdoor to indoor throughout the year for PT_B. The outside dry bulb temperature (DBT, T_o_) varied with a maximum diurnal variation of 20 °C and a minimum variation of 7 °C. The indoor air temperature (DBT, T_a_) variation is around 5 °C. Similarly, the variation of the MRT inside the space was around 2 °C and was maintained between 20 °C and 30 °C for this space.

**Figure 12:**
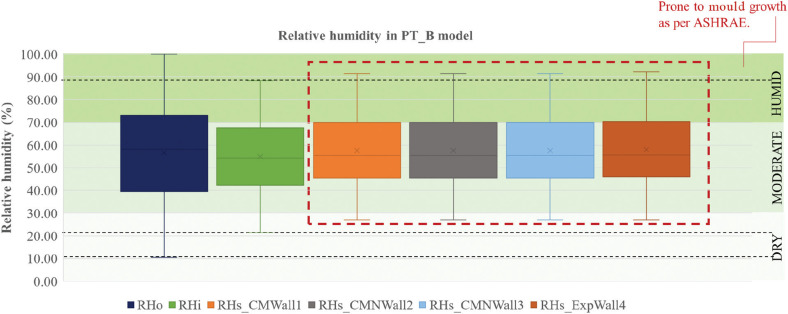
Indoor and surface RH levels in PT_B.

The RH of outdoor, indoor and four wall surfaces of PT_B are shown in Fig. 12. The outdoor maximum was 11.5% higher than the indoor maximum and the outdoor minimum was 11% lower than the indoor minimum. Also, the difference between the first quartile and third quartile was 33% for outdoor RH and 24% for indoor RH. It was observed that the surface RH levels were higher than the indoor RH levels. The inside surface RH (RHs) maximum was higher by 3% while the RHs minimum was higher by 7% when compared to the maximum and minimum values of the inside RH (RHi). To prevent mould growth, the American Society of Heating, Refrigerating and Air-Conditioning Engineers (ASHRAE) suggests that it is necessary to keep the spaces below 60% RH [37]. However, 25% of the surface RH values were >70%, leading to mould risk.

**Figure 13:**
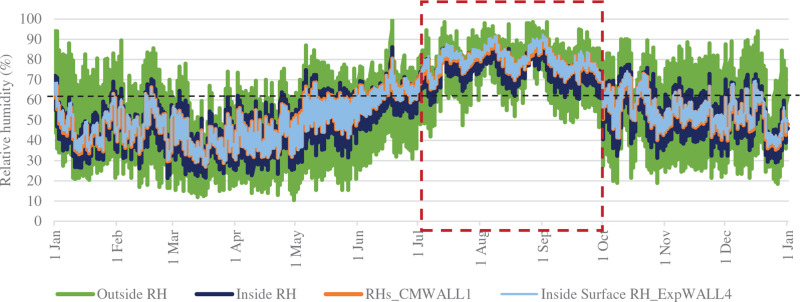
RH levels throughout the year in PT_B.

Figure 13 indicates varying RH levels over the year. During the monsoon months (from July to October), the indoor RH was always above 60%, while the surface RH of the walls was always above 68%. These are the months when the wall surfaces are highly prone to mould growth.

**Figure 14:**
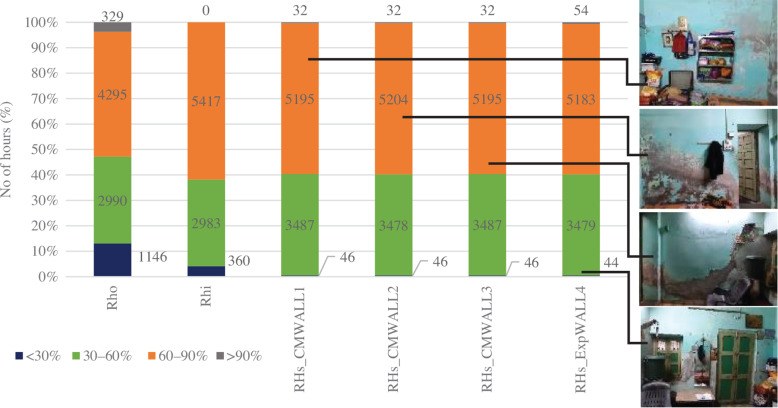
Hours corresponding to RH ranges over the year for space PT_B.

The percentage of hours when RH levels were favourable for mould growth is shown in Fig. 14. Out of 8760 hours, 32–54 hours were above 90% RH near the walls and 60% of the hours were between 60% and 90%, RH resulting in a risk of mould growth. Even though the simulation results show an equal percentage of high RH near the walls, the percentage of mould growth observed on-site over each wall was different. This could be because of the varied placement of furniture, openings and air velocity adjacent to each wall.

Another space PS_A showing mould on the walls was also simulated to check the hygrothermal model. However, for brevity, only important results are included for this space. In this case, the indoor RH maximum was lower than the outdoor maximum RH by 17%, and the maximum surface RH values were below 90%. The surface RH near the walls varied from 60% to 80% in the monsoon months (July–October). This period is favourable for mould growth. Through simulations of PS_A, the moisture buffering and indoor MRT were observed. The indoor RH was moderated, and the MRT was lower in these spaces. Lime-plastered spaces maintain MRT between 20 °C and 30 °C. The indoor RH levels were lower than the outdoor RH by 9–11%. Overall, simulations helped to predict the duration for which the walls would be exposed to high RH levels (>70%) that lead to mould growth. Thus, the EMPD model was able to predict conditions favourable for mould growth in the two surveyed spaces.

### Survey results

The observations from the surveyed Pol houses and residences are discussed in this section.

#### Pol houses

**Figure 15:**
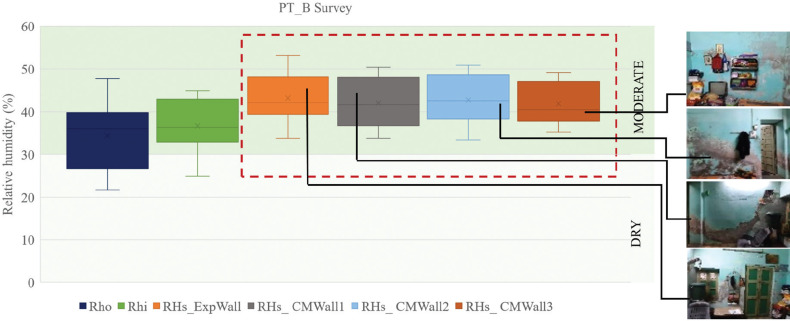
Onsite RH levels inside PT_B.

The Pol houses surveyed were categorised according to the coatings used over lime plaster. During the study period (December–March) the RH levels varied between 10% and 55% inside the studied spaces. The indoor minimum values of RH were higher by 2.1% and 1% in the case of lime-washed and nonporous painted walls. Slightly higher indoor RH levels could be due to indoor occupancy and moisture generation. To further examine the simulation observations for sample space PT_B, the onsite readings were analysed. In Fig. 15, the outdoor RH is compared with the indoor and surface RH levels from January to March.

Similar to the simulation results (refer to Fig. 12), surface RH levels were higher than the indoor RH levels. Fifty per cent of the indoor readings were higher than outdoor readings, indicating a humid indoor environment. The minimum values of surface RH were 9.8–12.4% higher than the minimum indoor RH. The maximum surface RH is 4.3% to 8.3% higher than the maximum indoor RH. If the above pattern is followed throughout the year, whenever the space RH goes above 60%, the surface RH will be around 65–70% RH. In the monsoon period, when the outside RH (RHo) levels are in the range of 80–95%, the surface RH of the walls could reach 95–97%. These RH levels are favourable for mould growth unless the moisture is removed from the surface.

#### Girikunj residence and RSR residence

**Figure 16:**
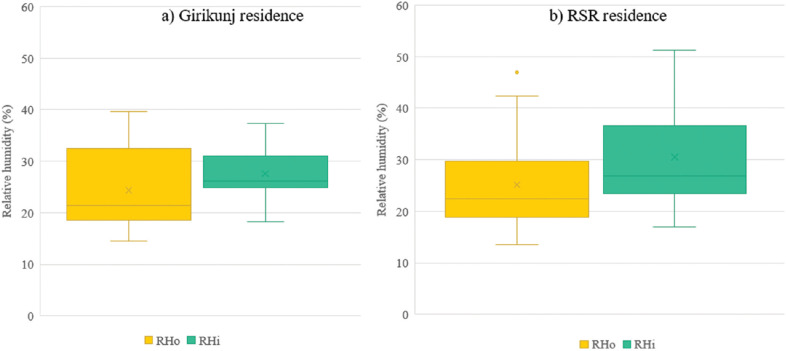
Indoor RH of different spaces.

For the Girikunj residence, the surveyed spaces included a basement and the topmost rooms exposed to the sun. Figure 16a shows the RH in the study period of March. The outdoor RH near Girikunj varied between 14.6% and 40%. The RH in indoor spaces varied between 18.2% and 37.4%. A difference of 3–4% was observed between the high and low values from outdoor to indoor. No water activity or occupancy (excluding the surveyor) was observed inside any of these spaces.

The topmost coating in the RSR residence is Venetian lime plaster, which is a smooth marble-like lime plaster that does not require any paint. Thirteen spaces on the ground floor were surveyed in the month of March. Figure 16b shows the indoor and outdoor RH levels. The mean of indoor RH was higher than that of outdoor by 4.7%. Mould growth was observed in one of the spaces (RJ_K). In this space, the indoor RH was higher by 8–14% and a maximum difference of 18% was observed between the surface RH and indoor RH. An adjacent space, RJ_J, was found to be free of mould. The indoor RH in RJ_J space was higher by 1–5% while the surface RH was higher than indoor RH by 5–9%. Thus, the correlation between higher surface RH and mould growth was observed through on-site surveys.

#### MRT observations

**Figure 17:**
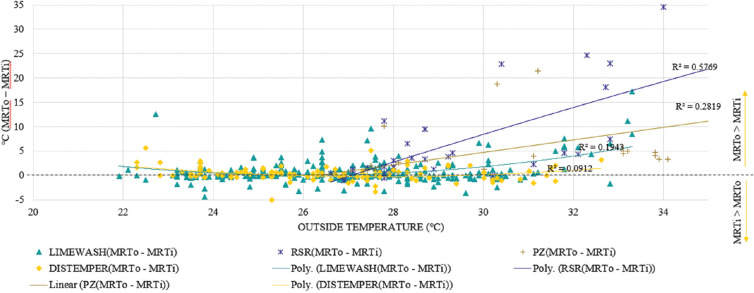
MRT variation with outdoor temperature for different spaces. MRTo, outside mean radiant temperature; MRTi, inside mean radiant temperature.

Figure 17 shows the difference between outdoor and indoor MRT for all the studied spaces. The MRT of space was calculated as per the formula given in [38]. With increasing outdoor temperatures, the difference increases for all the spaces except those having a nonporous coating over lime plaster. A positive difference shows that the indoor MRT is lower than the outdoor MRT. The differences were highest (indicated by a steeper trendline) for the RSR residence where lime plaster was exposed. The differences for Girikunj were lower than the RSR residence and least for Pol houses with limewash.

The indoor was considered to be comfortable when inside MRT (MRTi) was found to be lower than the outside MRT (MRTo). Based on the above criteria, it was observed that the spaces with nonporous paint were hot 30% of the time. Limewashed spaces in Pol houses remained hot for 18.5% of the time, while RSR and Girikunj residences stayed hot for 8.6% and 7.4% of the time, respectively. Thus, the RSR and Girikunj residences were comfortable for more than 90% of the time. Pol houses with limewash were comfortable for 81% of the time in March. These differences could be more in hotter months and need to be studied further by year-round measurements.

#### Mould risk observations

**Figure 18:**
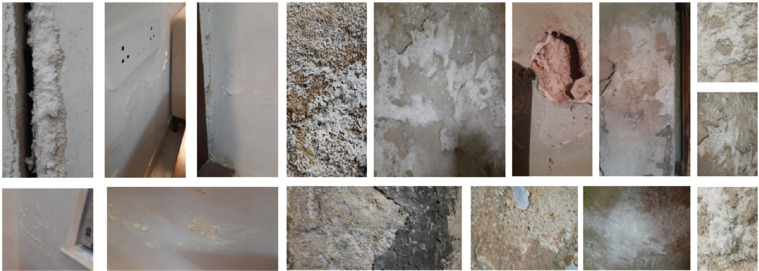
Onsite photos of different locations with mould growth.

From the surveys, it was observed that the hygrothermal behaviour of spaces varied with their characteristics. The spaces were identified based on the topmost finish of the wall surface layer, air velocity inside the space, occupancy, storage, the sunlight received and the type of activity happening in that space. Figure 18 shows photos of different spaces with visible mould growth.

**Figure 19:**
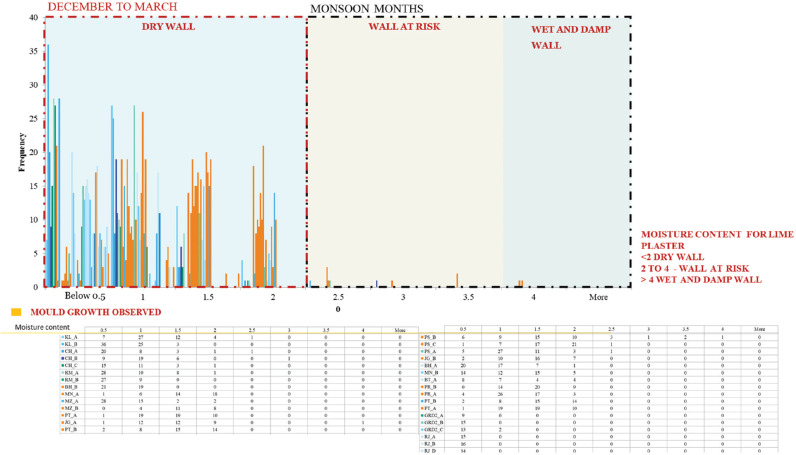
Moisture content of all walls surveyed between December 2019 and March 2020.

For further analysis, the moisture content of the walls (*μ*) was recorded and is shown in Fig. 19. The walls of the spaces where mould growth is observed are marked in orange. During the study period from December to March, all the walls were dry and showed low moisture content below 2%. However, mould growth was still observed on these walls. The mould would have grown during the past monsoon season from July to October when the RH levels were in excess of 90% for sustained periods. Factors such as damaged construction, the presence of water pipelines, low ventilation, etc., were other reasons for mould.

**Figure 20:**
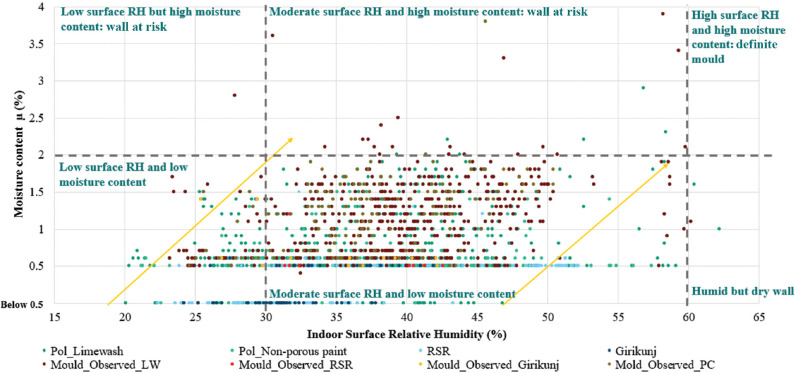
Moisture Content vs. surface relative humidity.

As the study was conducted in drier months there were hardly any walls having a moisture content of >2%. Nonetheless, mould was present on these walls, which can further be analysed by understanding the correlation of moisture content with the surface RH. In Fig. 20, the wall moisture content is plotted against the indoor surface RH. The points marked in orange, ochre, red and maroon represent the readings of walls having mould. The rest of the points are shown in blue. The graph is divided into six sections by referring to the characteristic curve of lime mortar in the moisture meter [39]. For surface humidity above 60% and moisture content above 2%, there are definite chances of mould. For RH below 30% and 1.5 moisture content, the conditions are dry enough to restrict mould. Walls with mould showed moisture content >0.5%.

In the case of Pol houses, most of the readings in Fig. 20 are populated in the section of moderate humidity and dry wall. These also include walls affected by mould due to the predated wet monsoon season. Thus, the poor performance of the building during monsoons or due to specific damage is evident. Points lying below 60% surface humidity and above 2% moisture content suggest mould growth due to the trapped moisture in the walls. Points in bright red are of the RSR residence, where the reason for the presence of mould was the existence of a water pipeline and high moisture source (pool) outside the space. In the Girikunj residence (marked in dark blue) the mould was observed in the basement and the space attached to the terrace. Due to leakage near the slab of the terrace during rain, the walls might have been damaged. There are numerous blue points observed towards the right of the graph, indicating well-ventilated and undamaged walls. Overall, a gradual shift towards the right is observed with the increase in indoor surface RH. This indicates the increase in moisture content with the increase in the indoor surface RH.

**Figure 21:**
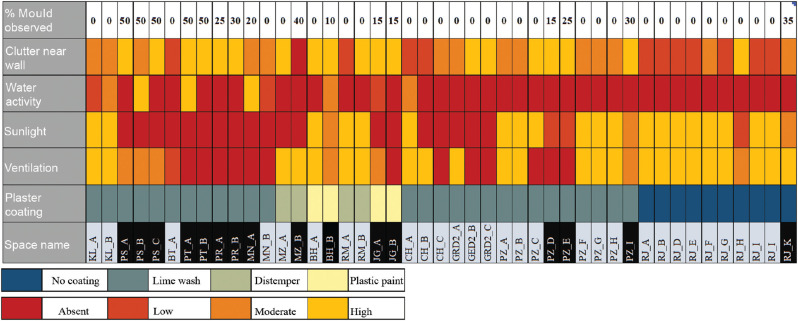
Space characteristics and mould growth relationship.

Figure 21 shows the mould scenario of each space with respect to its characteristics. It helps in identifying the strongest and most common factors that affect mould growth as observed and measured during the multiple site visits. The spaces highlighted in black are indicative of mould growth. All the spaces were further categorised and colour coded as per the following characteristics:

1. Plaster paint: coating used over the plaster-like lime wash paint, distemper paint (cement paint, made by mixing chalk, lime water and animal glue) [40], plastic emulsion paint or not coated. If the topmost coating is nonporous, the moisture is trapped inside, resulting in mould growth.
2. Ventilation: if the wall surfaces are not properly ventilated, the moisture inside the pores of the finishing layer does not evaporate and leads to mould growth.
3. Sunlight: sunlight plays a role in killing bacteria and keeping highly humid surfaces dry.
4. Water activity: high water activity, that is, activities such as washing, cleaning, etc., result in higher moisture inside the space.
5. Clutter near the wall (storage): more cluttered spaces create humid pockets inside the space. Clutter is considered low, moderate and high if the furniture and other items are blocking the walls by 30%, 60% or 70%. This was based on just visual observations.

The best and worst combination for predicting mould risk can be inferred from Fig. 21. For example, the worst-case scenario was observed in spaces PT_B and PT_A. More than 50% of the walls were densely covered with mould. PT_B has a good scope of ventilation, but the openings were always closed. So, it can be marked as not ventilated, with no sunlight, low water activity, moderate storage near walls and a limewash coating on the wall. Even though there was no water activity, other factors were dominating. The PT_A space was characterised by closed openings, no sunlight, high water activity, moderate storage near the walls and lime wash. In both cases, it was observed that due to a lack of ventilation and no sunlight the moisture was trapped inside the space, which led to mould growth. The best combination is where lime plaster is not coated or coated with limewash, and the spaces were well ventilated and sunlit. The surveys suggest that mould growth in lime plaster was observed if the moisture transfer is obstructed. Therefore, if lime-plastered surfaces are allowed to breathe in ventilated spaces mould growth can be avoided.

The number of studied spaces/rooms in Pols accounted for 57% and the spaces/rooms in the in the individual residences accounted for 43% of the spaces. In comparison to the Pol houses, the individual residences had better strategies such as air vents, daylight spaces and periodic maintenance. In the hotter month of March, they show better hygrothermal performance and were comfortable for more than 90% of the time. The indoors were cooler than the outdoor by 1 °C to 5 °C. The walls were drier and were recorded to be mostly below 0.5% of moisture content. The exposed lime plaster helped in moderating the indoor RH levels. The major issue observed in lime-plastered Pol houses was the lack of proper ventilation, sunlight and maintenance.

#### Mould observation under the microscope

**Figure 22:**
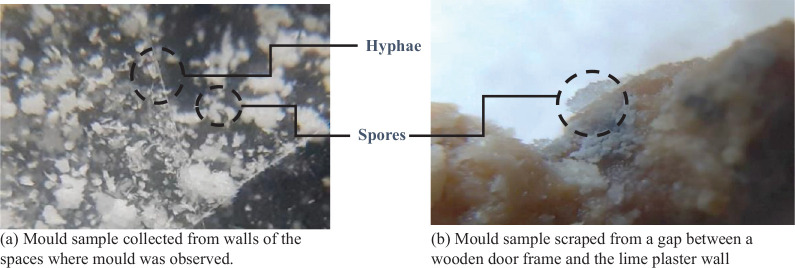
Mould samples under the 40× simple microscope.

Samples were collected from the site to verify the presence of mould on the walls. The most common type of mould that is formed over lime plaster was observed. Figure 22 shows the collected samples as observed under a 40× simple microscope. In Fig. 22a, the thread-like filaments are the hyphae of the mould structure. The small granules are likely to be spores. Figure 22b shows a sample scraped from a gap between a wooden door frame and a lime plaster wall. This type of mould is different from the one observed on the walls having lime plaster. Observation under a high-resolution compound microscope will be required to identify the type of mould.

#### Studying the onset of mould growth on lime plaster samples

**Figure 23:**
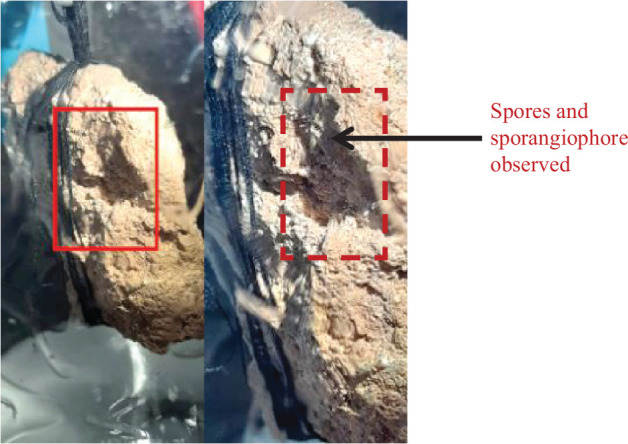
Mould growth observed under 96% RH.

All the samples of lime plaster weighed 20 g and had an initial moisture content of 0.6%. Of the four containers, the one containing potassium sulphate (96% RH) had early signs of mould growth. The other three containers (having RH of 75%, 80% and 86%) showed no visible mould growth until the third week. However, at 96% RH mould growth was visibly seen on the lime plaster sample within 3 weeks (see Fig. 23). Thus, a favourable environment for mould growth is created if the surface RH in the space is >95%. This observation can be related to the conditions on site where mould is observed on the walls considering that the humidity near those walls has been consistently high for some time.

## Conclusion

In this work, the hygrothermal performance of lime plaster in naturally ventilated spaces was studied and co-related with mould risk. The survey of the lime-plastered spaces was carried out between December 2019 and March 2020. In relatively dry weather conditions, surface RH levels of the studied spaces were higher than the outdoor RH. The indoor RH levels are expected to rise further in the wet monsoon period and therefore are prone to mould growth. The mould growth was not observed in spaces that were well maintained (uncluttered), well-ventilated and sunlit. Nonporous coatings on plastered surfaces led to mould growth. Thus, it is important to maintain a breathable wall assembly and adequate ventilation to facilitate moisture transfer across the building envelope. Spaces finished with a lime wash and exposed lime plaster also showed lower indoor MRT than spaces with nonporous coatings.

Hygrothermal simulations with the EMPD model were able to capture moisture buffering in different materials. Hygrothermal simulations carried out with the EMPD model were able to predict the indoor conditions which might result in mould formation. Thus, mould growth risk on different wall surfaces can be identified. However, more numerical analysis with hygrothermal properties of typical lime plaster used in India needs to be carried out for realistic predictions. Walls with high surface RH were susceptible to mould risk if exposed to indoor RH levels >60% for more than 4 weeks. Experiments with lime plaster samples showed visible mould growth in 3 weeks under an environment with a RH of around 95%. By combining the above observation with the simulations, the chances of mould growth in a space can be predicted. The performance gap between a simulation and an actual case can also be narrowed down for better predictions if the simulations are carried out in collaboration with survey observations.

Overall, lime plaster is a sustainable and low embodied energy material with good moisture buffering capacity. It can moderate indoor RH levels and lead to lower MRT. Although it is prone to mould growth in high humidity conditions, avoiding nonporous coatings and maintaining adequate ventilation can reduce mould risk. Especially suited for historic buildings, lime plaster should also be considered in contemporary building practices.

Future work on identifying the composition of lime plaster composition to relate it to mould growth is necessary. Hygric property characterisation is also required for carrying out detailed hygrothermal simulations for predicting mould formation. Finally, a year-round study will give a comprehensive picture of the interrelated parameters responsible for mould growth.

## Data Availability

The datasets generated during and/or analysed during the current study are available from the corresponding author on reasonable request.

## Note

1 Ahmedabad weather data. https://energyplus.net/weather-location/asia_wmo_region_2/IND/IND_Ahmedabad.426470_ISHRAE.

## Appendix

**Table A1.** Characteristics of studied spaces

Space name	Wall details	Mould growth observed	Function	Coating over plaster	Volume	Occupancy	Age of buildings (yrs) (estimated)
KL_A	North and west wall exposed	No visible mould growth	Living area	Lime wash	34.52	1	100-300
KL_B	South and west wall exposed	Outside exposed wall	Kitchen	Lime wash	36.62	0	100-300
PS_A	North wall exposed	No visible mould growth	Living area	Lime wash	69.01	3	100-300
PS_B	Open to sky	On 1 common wall	Kitchen	Lime wash	120.06	0	100-300
PS_C	No exposed wall	On all walls	Store room	Lime wash	21.09	0	100-300
BT_A	North wall exposed	No visible mould growth	Bedroom	Lime wash	32.10	4	100-300
PT_A	South and west wall exposed	Inside and outside all walls	Common area	Lime wash	33.68	0	100-300
PT_B	North wall exposed	On all walls	Bedroom	Lime wash	23.32	0	100-300
PR_A	East wall exposed	On all walls	Store room	Lime wash	72.90	0	100-300
PR_B	No exposed wall	On all walls	Store room	Lime wash	25.80	0	100-300
MN_A	South wall exposed	On common walls	Living area	Lime wash	25.26	3	100-300
MN_B	North wall exposed	No visible mould growth	Kitchen	Lime wash	34.52	1	100-300
MZ_A	East wall exposed but shaded	No visible mould growth	Small scale industry activity	Distemper	28.92	3	100-300
MZ_B	No exposed wall	On both the walls	Passage	No coating	7.51	0	100-300
BH_A	West wall exposed	On east wall	Living area	Plastic paint	41.10	3	100-300
BH_B	North and west wall exposed	On east wall	Kitchen	Plastic paint	31.50	1	100-300
RM_A	East wall exposed	No visible mould growth	Living area	Distemper	39.30	4	100-300
RM_B	North wall exposed	No visible mould growth	Bedroom	Distemper	32.10	1	100-300
JG_A	North and east wall exposed	On all walls	Common area	Plastic paint	70.00	0	100-300
JG_B	No exposed wall	On all walls	Store room	Plastic paint	45.00	0	100-300
CH_A	North wall exposed	No visible mould growth	Kitchen	Lime wash	26.19	1	100-300
CH_B	No exposed wall	No visible mould growth	Bedroom	Lime wash	52.96	1	100-300
CH_C	No exposed wall	No visible mould growth	Store room	Lime wash	6.28	0	100-300
GRD2_A	East wall exposed	No visible mould growth	Dining area	Lime wash	37.92	0	100-300
GED2_B	West wall exposed	No visible mould growth	Store room	Lime wash	18.79	0	100-300
GRD2_C	East wall exposed	No visible mould growth	Store room	Lime wash	22.75	0	100-300
PZ_A	South and west wall exposed	No visible mould growth	Living area	Lime wash	210.77	0	50-80
PZ_B	South and west wall exposed	No visible mould growth	Bedroom	Lime wash	44.21	0	50-80
PZ_C	South and west wall exposed	On common walls	Basement store room	Lime wash	45.26	0	50-80
PZ_D	South and west wall exposed	No visible mould growth	Basement store room	Lime wash	110.52	0	50-80
PZ_E	South and west wall exposed	On 2 common walls	Basement store room	Lime wash	94.33	0	50-80
PZ_F	South and west wall exposed	No visible mould growth	Common area	Lime wash	107.28	0	50-80
PZ_G	South and west wall exposed	No visible mould growth	Bedroom	Lime wash	49.50	0	50-80
PZ_H	South and west wall exposed	No visible mould growth	Bedroom	Lime wash	113.83	0	50-80
PZ_I	South and west wall exposed	On exposed walls	Terrace common area	Lime wash	250.00	0	50-80
RJ_A	South and west wall exposed	No visible mould growth	Living area	No coating	196.00	0	10-15
RJ_B	South and west wall exposed	No visible mould growth	Dining area	No coating	126.60	2	10-15
RJ_D	South and west wall exposed	No visible mould growth	Living area	No coating	189.00	2	10-15
RJ_E	South and west wall exposed	No visible mould growth	Bedroom	No coating	69.60	0	10-15
RJ_F	South and west wall exposed	No visible mould growth	Store room	No coating	33.60	0	10-15
RJ_G	South and west wall exposed	No visible mould growth	Store room	No coating	105.00	0	10-15
RJ_H	South and west wall exposed	No visible mould growth	Store room	No coating	47.20	0	10-15
RJ_I	South and west wall exposed	No visible mould growth	Living area	No coating	183.41	1	10-15
RJ_I	South and west wall exposed	No visible mould growth	Entrance lobby	No coating	17.92	0	10-15
RJ_K	South and west wall exposed	Inside and outside exposed walls	Living area	No coating	73.41	1	10-15
